# Non-coding RNAs and HIV: viral manipulation of host dark matter to shape the cellular environment

**DOI:** 10.3389/fgene.2015.00108

**Published:** 2015-03-26

**Authors:** Samantha Barichievy, Jerolen Naidoo, Musa M. Mhlanga

**Affiliations:** ^1^Gene Expression and Biophysics Group, Synthetic Biology Emerging Research Area, Council for Scientific and Industrial Research, PretoriaSouth Africa; ^2^Discovery Sciences, Research & Development, AstraZeneca, MölndalSweden; ^3^Gene Expression and Biophysics Unit, Instituto de Medicina Molecular, Faculdade de Medicina da Universidade de Lisboa, LisbonPortugal

**Keywords:** microRNAs, HIV-1, long non-coding RNA, immune evasion, host–pathogen interactions, apoptosis, double strand breaks

## Abstract

On October 28th 1943 Winston Churchill said “we shape our buildings, and afterward our buildings shape us” (Humes, 1994). Churchill was pondering how and when to rebuild the British House of Commons, which had been destroyed by enemy bombs on May 10th 1941. The old House had been small and insufficient to hold all its members, but was restored to its original form in 1950 in order to recapture the “convenience and dignity” that the building had shaped into its parliamentary members. The circular loop whereby buildings or dwellings are shaped and go on to shape those that reside in them is also true of pathogens and their hosts. As obligate parasites, pathogens need to alter their cellular host environments to ensure survival. Typically pathogens modify cellular transcription profiles and in doing so, the pathogen in turn is affected, thereby closing the loop. As key orchestrators of gene expression, non-coding RNAs provide a vast and extremely precise set of tools for pathogens to target in order to shape the cellular environment. This review will focus on host non-coding RNAs that are manipulated by the infamous intracellular pathogen, the human immunodeficiency virus (HIV). We will briefly describe both short and long host non-coding RNAs and discuss how HIV gains control of these factors to ensure widespread dissemination throughout the host as well as the establishment of lifelong, chronic infection.

## Long Non-Coding RNAs: An Added Layer of Complexity in Gene Regulation

Only 2% of the metazoan genome encodes protein, yet more than 50% is transcribed and our knowledge is limited regarding these transcripts that function in the absence of protein production. In fact, stable non-coding RNA transcripts have been referred to as ‘dark matter’ within the cellular environment (Yamada et al., 2003). Despite improvements in the human draft genome sequence, non-coding RNAs remain difficult to define and thus quantify (Ponting and Belgard, 2010; Saxena and Carninci, 2011). Numerous evolutionary and sequencing studies have revealed that non-coding RNAs could be expressed at up to 20-fold excess compared to their protein-coding counterparts, and are highly conserved (Ponting, 2008; Nagano and Fraser, 2011). Recently, ‘dark matter’ has been confined to those transcripts that constitute the biggest class of non-protein-coding RNAs, so-called long non-coding RNAs (lncRNAs), which lack an open reading frame and are longer than 200 nucleotides (Derrien et al., 2012). Approximately 10 000 lncRNAs have been annotated in humans (Wilusz et al., 2009; Djebali et al., 2012) and additional mammalian catalogs are being continually refreshed (Guttman et al., 2009; Marques and Ponting, 2009; Jia et al., 2010; Ørom et al., 2010; Cabili et al., 2011).

The majority of lncRNAs described to date are independent transcriptional units with canonical splice sites and alternatively spliced variants. However, they tend to have only two exons that are also slightly longer than their protein-coding counterparts (Derrien et al., 2012). While the majority of lncRNAs are located between protein-coding genes (termed long intergenic RNAs or lincRNAs; Khalil et al., 2009), the remaining lncRNAs span or intersect both exonic and intronic regions of various protein-coding genes (Derrien et al., 2012). Notably, lncRNAs show a striking degree of tissue-specific expression as well as co-expression with neighboring genes (Cabili et al., 2011; Derrien et al., 2012), and are highly conserved across primates (Derrien et al., 2012). In addition, many lncRNAs display chromatin signatures typically associated with promoters and transcribed regions (histone 3 lysine 4 tri-methylation, H3K4me3, and histone 3 lysine 36 tri-methylation, H3K36me3, respectively; Guttman et al., 2009; Derrien et al., 2012). Given the intimate connection to these epigenetic marks, it is unsurprising that lncRNAs are preferentially enriched within chromatin and nuclear RNA fractions (Mondal et al., 2010). Their nuclear location also hints at lncRNA function in modulating protein-coding-gene activity.

Generally, the observation that lncRNAs are in close proximity to known protein-coding genomic regions (van Bakel et al., 2010; Cabili et al., 2011) and are particularly concentrated near transcription factors (Guttman et al., 2009; Ponjavic et al., 2009), suggests a role in gene regulation. Indeed, 100 lncRNAs were found to be under the control of specific key transcription factors including p53, NFκβ, Sox2, and Nanog (Guttman et al., 2009). In a separate set of studies, four lncRNAs (Xist, Air, Kcnq1ot1, HOTTIP) were robustly shown to regulate transcription of numerous target genes through epigenetic modifications (Brown et al., 1991; Penny et al., 1996; Sleutels et al., 2002; Pandey et al., 2008; Wang et al., 2011). By altering the histone proteins around which DNA is wrapped, lncRNAs can tune expression of the associated genes through activation or repression of the chromatin (Flynn and Chang, 2012). Chromosomal looping has also been suggested to play a part in lncRNA function as many appear to be transcribed from enhancer regions (Ørom et al., 2010; Wang et al., 2011). These latter transcriptional control elements are capable of activating gene expression independent of their location or distance. Furthermore, while the exact mechanisms of gene regulation by lncRNAs remain largely obscure, their ability to control host transcription provides a critical point of manipulation for pathogens. Although limited in genome size, pathogens specifically and directly alter host gene expression profiles in their favor, and obligate parasites such as HIV, seem to be particularly adept at this.

## Long Non-Coding RNAs and Proteins: Functioning together *In Cis* and *In Trans*

To date, only a small number of lncRNAs have been functionally characterized. This is testament to the difficulty of detecting these RNA species which tend to be expressed at very low levels (Ravasi et al., 2006; Cabili et al., 2011), as well as the use of direct perturbation experiments required to identify their functional roles. Typically gain- or loss-of-function studies are used but often the choice of which phenotype to investigate remains unclear (Willingham et al., 2005). Despite these challenges, lncRNAs have been shown to regulate gene expression from the level of chromatin modification and transcription through to RNA maturation, transport and translation (Wapinski and Chang, 2011). In addition, lncRNAs seem to function both *in cis*, by exerting their effect(s) on a neighboring gene located on the same allele as the transcribed lncRNA, or *in trans* when the lncRNA and target gene are not on the same allele (Guttman and Rinn, 2012). Notably, lncRNAs all seem to function via their interaction with one or more protein partners (reviewed in Nagano and Fraser, 2011; Wang and Chang, 2011; Guttman and Rinn, 2012; Cech and Steitz, 2014). Together these ncRNA-protein complexes perform a myriad diverse functions with a surprising degree of complexity. While the details of each mechanism are beyond the scope of this review and have been covered elsewhere (Nagano and Fraser, 2011; Wang et al., 2011; Guttman and Rinn, 2012; Kornienko et al., 2013), some lncRNA-protein interactions are noteworthy because they pertain to HIV.

## Long Non-Coding RNAs and HIV: Viral Manipulation at the Heart of Gene Regulation

As an intracellular pathogen, HIV relies on host cellular machinery to complete its life cycle. Integral to this is the modulation of host gene expression to ensure a co-ordinated regulation of pro- and anti-viral host factors (Strebel et al., 2009; Rasaiyaah et al., 2013). Given that the virus specifically carves out the transcriptional status of infected host cells, and that lncRNAs regulate transcription, it is unsurprising that HIV directly manipulates these specific host factors. As a retrovirus, HIV converts its RNA genome to a DNA copy that is then integrated randomly into host chromatin. This action induces DNA damage in the host genome, alters chromatin structure, triggers innate immunity and ultimately ensures latency and chronic infection for the virus. Multiple facets of gene regulation are involved in each of these steps but to date, only a couple of HIV-lncRNA interactions have been described (Zhang et al., 2013; Barichievy et al., submitted). In each case, host lncRNAs that regulate innate immunity or the cellular response to DNA damage are manipulated by HIV. However, given the complexity of factors involved in gene regulation, it is likely that more HIV-host lncRNA interactions will be described.

### HIV and NEAT1

The mammalian nucleus contains many distinct structures including nearly 10 different nuclear bodies (Mao et al., 2011). One of these structures, the paraspeckle, forms around the nuclear paraspeckle assembly transcript 1 lncRNA, NEAT1 (Hirose et al., 2014). Within paraspeckles, NEAT1 modulates cell survival in response to stress by repressing transcription of several genes via sequestering specific proteins into the paraspeckle (Hirose et al., 2014; Imamura et al., 2014). One such host protein, splicing factor proline/glutamine rich (SFPQ), is sequestered by NEAT1 thereby releasing repression of the cytokine interleukin-8 (IL8; Imamura et al., 2014). The activation of IL8 is critical for the innate immune response, particularly following viral infection. Indeed, the interplay of NEAT1 and SFPQ regulates several antiviral innate immunity genes in response to influenza and herpes simplex viruses (Imamura et al., 2014). In HIV-infected CD4 T cells, NEAT1 has been shown to increase HIV expression by enhancing the nuclear export of viral mRNAs, although the molecular mechanism was not uncovered (Zhang et al., 2013). However, as NEAT1 also represses the RNA-specific adenosine deaminase B2 (ADARB2) gene, thereby controlling nucleocytoplasmic transport of ADAR-sensitive mRNAs (such as HIV transcripts), it is tempting to speculate that the virus manipulates NEAT1 to control innate immunity (via SFPQ) as well as post-transcriptional modulation of viral mRNAs (via ADARB2). Whether the virus interacts with NEAT1 or its protein binding partners is unclear, however, by targeting a single lncRNA involved in innate immunity, HIV ensures the cellular environment favors the virus.

### HIV and lincRNA-p21

Human immunodeficiency virus replication can only occur following successful integration of an HIV proviral genome within a host chromatin region that is conducive to gene expression (Lusic and Giacca, 2014). The virus is thus sensitive to the temporal and spatial dynamics of host chromatin architecture, and the establishment of HIV latency is intimately connected to this. Another pivotal characteristic of successful HIV replication underlies the integration event itself. Viral integrase is responsible for cleaving the host DNA and enabling integration of the proviral genome (Craigie and Bushman, 2012). Inherent in this action is the generation of a double strand break (DSB) within the cellular chromatin, which is the most detrimental form of DNA lesion for mammalian cells to undergo (Jackson and Bartek, 2009). As there is no intact complementary strand to serve as a template for repair, DSBs are poorly tolerated (Khanna and Jackson, 2001) with a single DSB sufficient to kill eukaryotic cells if it inactivates an essential gene (Rich et al., 2000). Given the potential severity of uncorrected DSBs, metazoan cells have evolved sensitive mechanisms to detect the damage (Hartlerode and Scully, 2009). The tumor suppressor protein p53 is a core transcription factor that plays a central role in the response to DNA damage (Meek, 2004). Activation of p53 leads to apoptosis, senescence or cell-cycle arrest (Zhou and Elledge, 2000). Cell-cycle arrest promotes survival by permitting time for the DNA damage to be repaired, while both senescence and apoptosis are terminal outcomes for the cell (Riley et al., 2008). As infecting retroviruses cannot co-ordinate the number of integration events and consequent DSBs per host cell, they would need to either mask the DSB from the cell or carefully orchestrate any cellular response to the DSB to avoid triggering apoptosis. Furthermore, to induce a DSB yet ensure survival, retroviruses must take control of prosurvival mechanisms and suppress activation of proapoptotic genes.

It is now understood that p53 outsources a critical portion of the apoptotic transcriptional response to a long intergenic non-coding RNA (Huarte et al., 2010). In response to DNA damage, p53 transcriptionally activates lincRNA-p21 which, together with a nuclear-localized protein binding partner hnRNP-K, orchestrates the apoptotic trigger by specifically repressing the transcription of prosurvival p53 target genes *in cis* (Huarte et al., 2010) and *in trans* (Dimitrova et al., 2014). One of these targets is MAP2K1, the primary kinase involved in phosphorylating ERK2 which functions in normal cells to ensure survival (Chang and Karin, 2001). In healthy cells, p53 is negatively regulated via HDM2-mediated ubiquitination and as a p53 transcription co-factor, hnRNP-K is similarly negatively regulated (Moumen et al., 2005; Enge et al., 2009). Thus p53-transcribed genes, including lincRNA-p21, are not expressed in healthy cells. Concurrently, activated ERK2 phosphorylates hnRNP-K thereby ensuring cytoplasmic accumulation of the latter protein (Habelhah et al., 2001) and preventing its association with lincRNA-p21. In addition, healthy cells further negatively regulate lincRNA-p21 via the action of nuclear HuR/ELAV1 by destabilizing the lincRNA through the action of Ago2 and let-7 (Yoon et al., 2012). The combined effects of these intersecting pathways ensures cellular survival. In contrast, DNA damage such as DSBs results in alternative modifications of p53 thereby negating HDM2 regulation (Enge et al., 2009). ERK2 is also not activated and thus hnRNP-K relocates to the nucleus where it can act with p53 to transcribe lincRNA-p21 (Moumen et al., 2005). As a complex, hnRNP-K and lincRNA-p21 then inactivate a suite of prosurvival genes leading to apoptosis (Huarte et al., 2010). Our most recent data show that HIV specifically and deliberately alters lincRNA-p21 function to mask integration-induced DSBs and gain control of the MAP2K1/ERK2 survival cascade (Barichievy et al., submitted).

**FIGURE 1:**
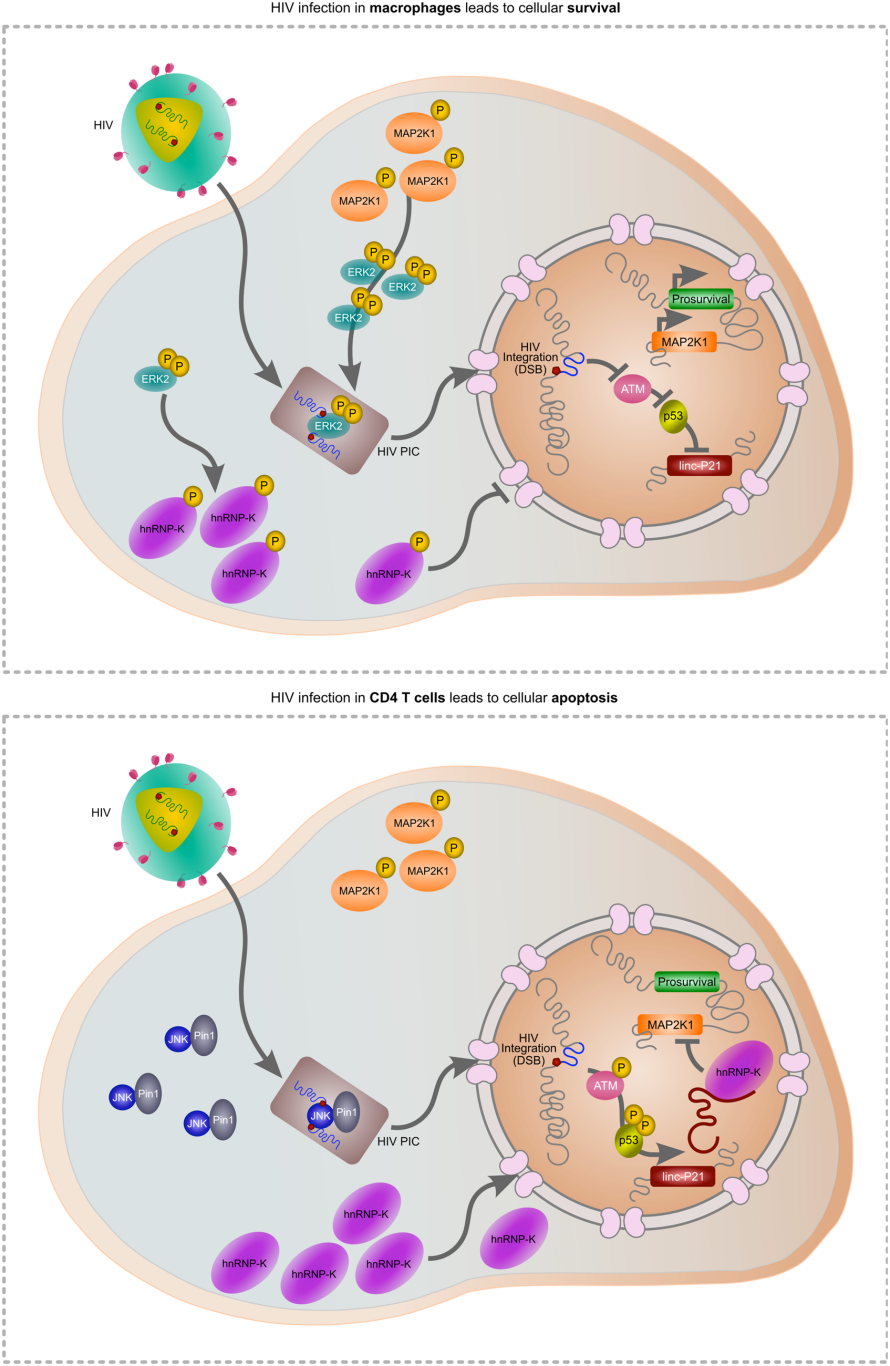
Hypothetical model of HIV-mediated manipulation of lincRNA-p21 to evade cellular apoptosis. During HIV infection of macrophages (upper panel), MAP2K1-activated ERK2 is incorporated into the pre-integration complex (PIC) enabling HIV integration. Active ERK2 also leads to the cytoplasmic accumulation of hnRNP-K thereby preventing its interaction with lincRNA-p21 in the nucleus. As p53 is not activated, lincRNA-p21 is not transcribed and cannot complex with hnRNP-K to suppress prosurvival genes including MAP2K1. In CD4 T cells, ERK2 expression is down-regulated and thus unavailable for incorporation in the PIC. JNK and Pin1 mediate integration but as the virus does not mediate control over ERK2, hnRNP-K can translocate to the nucleus, complex with p53-transcribed lincRNA-p21 and suppress prosurvival genes including MAP2K1, leading to apoptosis.

As HIV must induce a DSB during integration, apoptosis is a likely outcome of infection. Indeed, the progressive loss of CD4 T cells is a prognostic marker of disease, driven in part by integration (Cooper et al., 2013), but also due to abortive infection (Doitsh et al., 2010). Widespread dissemination of the virus throughout the host is facilitated by macrophages which are also readily infected by HIV. In contrast to CD4 T cells, macrophages are spared from TRAIL-induced apoptosis (Swingler et al., 2013) but more intriguingly, HIV is able to selectively impair apoptosis in macrophages by controlling MAP2K1/ERK2 and lincRNA-p21 (Barichievy et al., submitted). Activated ERK2 is required for successful HIV integration in macrophages and forms part of the pre-integration complex (PIC; Jacque et al., 1998; Bukong et al., 2010). As HIV gains control of this host protein prior to the integration event itself, the subsequent DSB can be masked. Indeed, our recent data show that HIV integration does not activate ATM autophosphorylation or downstream activation of apoptosis-specific marks on p53, and lincRNA-p21 is thus not transcribed by p53 (Barichievy et al., submitted). Key to these events is viral control of ERK2 and its upstream kinase MAP2K1, as inhibitors of these host factors results in apoptosis only in the presence of HIV. Importantly, MAP2K1 is a target of lincRNA-p21 thereby providing a connection between cell survival and apoptosis. Another consequence of viral control over MAP2K1/ERK2 is that hnRNP-K remains in the cytoplasm and is thus unavailable for its pro-apoptotic binding partner lincRNA-p21 (Barichievy et al., submitted). Nutlin3 can be used to overcome the HIV-induced nuclear entry block of hnRNP-K, and apoptosis does then occur (Barichievy et al., submitted). Notably, HIV integration in CD4 T cells is facilitated by JNK and Pin1 as opposed to ERK2 (Manganaro et al., 2010), possibly because ERK2 expression is switched off following differentiation in these cells (Fischer et al., 2005; Chang et al., 2012). The intimate connection between cell survival and apoptosis at the point of lincRNA-p21 and ERK2 thus possibly only occurs in macrophages, and it seems that HIV has evolved a pivotal mechanism to exploit this interaction in favor of viral survival (**Figure 1**).

## The Opposite of Long is Short: microRNAs in Transcriptional Regulation

The dark matter of the nucleus encompasses both short and long non-coding RNAs. The former include microRNAs (miRNAs) which are small endogenous non-coding RNA transcripts (∼22 nucleotides in length) that regulate host gene expression as part of the RNA interference (RNAi) pathway (Fire et al., 1998; Elbashir et al., 2001). The first miRNA was identified in 1993 in *Caenorhabditis elegans* by Ambros and colleagues, but the field has since exploded with the use of more recent technologies, including deep sequencing (Lee et al., 1993). There are currently over 2500 annotated human miRNAs, more than double the number that were identified only 2 years ago (Kozomara and Griffiths-Jones, 2014). Furthermore, it has been estimated that more than 60% of all human genes are regulated by endogenous miRNAs (Friedman et al., 2009), highlighting the pivotal role of these ncRNAs in cellular transcription. Indeed, a single miRNA can regulate multiple mRNA molecules that can in turn also be acted upon by numerous miRNAs (Barbato et al., 2009; Sashital and Doudna, 2010). The non-orthogonal nature of miRNA-mRNA interactions means that adjustments to expression levels of even a few miRNAs can facilitate the rapid and synchronized shift in the expression levels of hundreds of genes (Davis and Hata, 2009). Thus, the endogenous miRNA pathway represents a highly efficient system to simultaneously fine-tune the expression of numerous genes as well as modulate specific functional pathways (Hwang and Mendell, 2007), including innate immunity and host–pathogen interactions (Sayed and Abdellatif, 2011; Seddiki et al., 2014). Here we will discuss cellular miRNAs that are perturbed following HIV infection, then shift focus on to specific host miRNAs that act either directly or indirectly on HIV to regulate viral replication, and look at counter strategies used by the virus to evade RNAi.

## The Impact of HIV Infection on Host miRNA Expression Profiles

Over the last decade a number of studies have reported a general perturbation of the host miRNA landscape following HIV infection (Klase et al., 2012; Swaminathan et al., 2014). An early microarray-based study that had utilized HeLa cells transfected with an infectious pNL4-3 clone, showed the virus down-regulated 312 host miRNAs, while ensuring none were up-regulated (Yeung et al., 2005). These findings were then contradicted in a study that utilized the same microarray approach, applied to Jurkat cells and HIV-infected patient-derived PBMCs, to show that the miR-17/92 cluster was down-regulated and 11 host miRNAs were up-regulated (Triboulet et al., 2007). In addition, this study revealed that host miR-122, miR-370, miR-373^∗^ and miR-297 were exclusively expressed in HIV-infected cells up to 42 days post-infection, supporting the hypothesis that HIV uses these host factors to modulate functional cellular pathways in favor of the virus. Indeed, in a more recent study that evaluated the expression levels of 702 host miRNAs as well as 25, 000 mRNA species in response to HIV infection, *in silico* analysis revealed perturbations in the apoptosis, MAPK, T cell receptor and Wnt signaling pathways (Gupta et al., 2011). Thus it is clear that HIV disturbs cellular miRNA expression during infection.

While HIV-mediated modulation of the host miRNA landscape seems highly selective, the poor concordance between different studies has made tabulating a reliable list of such miRNAs difficult. In a recent publication, Whisnant et al. (2013) sought to address these conflicts by incorporating data from different cell types, viruses, and sequencing methods. In this study, BAL and pNL4-3 infection in TZM-bl cells, C8166 T cells, and PBMCs, only altered the expression levels of a few host miRNAs 72 h post-infection. A single miRNA, miR-30b-5p, was up-regulated in BAL-infected PBMCs but not in similar cells infected with pNL4-3. Furthermore, in contrast to previous data from PBMCs (Triboulet et al., 2007), no significant change in the miR-17/92 cluster was observed regardless of the cell type/viral strain used (Whisnant et al., 2013). While the latter findings contradict previous studies (Triboulet et al., 2007; Gupta et al., 2011; Sun et al., 2012), the different time points and half-lives of the relevant cellular cultures used to assess host miRNA expression profiles differed between the data sets. Timing seems to be a key factor to consider. Indeed, a recent study that utilized small RNA seq at 5, 12, and 24 h post-infection in Sup-T1 cells, showed discrete and temporal effects on the expression levels of 15 host miRNAs (Chang et al., 2013). A distinct subset was suppressed at 5 and 12 h, yet recovered by 24 h post-infection. An overlay with corresponding mRNA expression data revealed that T cell activation and cell cycle pathways were being similarly differentially regulated.

A comparison of the findings from the Chang and Whisnant groups revealed that only two miRNAs, miR-143-3p and miR-10a-5p, exhibited similar trends of suppressed expression. Thus, even after factoring in cell type variation and the confounding effects of time, it is clear that the impact of experimental parameters restrains comparisons between such datasets. Given this, elite controllers, may provide the most informative data in this arena. Elite or viremic controllers are individuals who are able to naturally restrict HIV replication to undetectable levels (<50 viral copies/mL; Blankson, 2010). The exact mechanisms by which they are able to effect this control over HIV replication are not understood. However, as host miRNAs inherently have antiviral properties, the possibility that they mediate some aspect of the ‘elite control’ remains an area of great interest. Several studies that investigated miRNA expression profiles in elite controllers revealed that miR-150 and miR-125b, miR-31 and miR-31^∗^ were consistently suppressed in these patients (Houzet et al., 2008; Witwer et al., 2012; Reynoso et al., 2014). In addition, miR-29b-3p, miR-33a-5p and miR-146a-5p were elevated in elite controller samples compared to chronically infected patients (Reynoso et al., 2014). Intriguingly, miRNA pathway analysis revealed the Toll-like receptor signaling pathway to be regulated by these three miRNAs (Vlachos et al., 2012). The central role of this host pathway in restricting pathogens is certainly in line with the ability of elite controllers to control HIV replication.

While all of studies touched upon here have reported perturbations in host miRNA expression profiles in response to HIV infection, there is only modest consensus on both the degree to which the virus is able to manipulate global miRNA expression, as well as the specific miRNAs involved. When considering the combined implications of these studies, a few salient points are noteworthy: (i) the effect of HIV infection on host miRNA expression is highly dependent on experimental variables including cell type, HIV variant, and the method of quantification (Yeung et al., 2005; Triboulet et al., 2007; Chang et al., 2013; Whisnant et al., 2013); (ii) while the individual miRNAs identified between studies may differ it is clear that miRNA dysregulation is targeted to specific functional pathways important for HIV replication (Gupta et al., 2011; Reynoso et al., 2014); (iii) the effect of HIV infection on host miRNA expression is dynamic and may orchestrate a complex series of temporally sensitive molecular events (Chang et al., 2013; Whisnant et al., 2013); and (iv) the discrimination between viral and host-driven effects on miRNA expression are important but not yet well-described and thus further attention.

## Host miRNAs that Act on HDFs to Affect HIV (Indirect Effects on Viral Replication)

A number of host miRNAs have been identified as modulators of HIV replication and elicit their effects either via a direct interaction with viral products, or indirectly via the regulation of viral host dependency factors (HDFs). Direct interaction with viral proteins generally leads to decreased HIV replication, while the targeting of HDFs can lead to either increased or decreased viral replication. A summary of these findings is in **Table 1** and we expand on some of the more recent observations here, beginning with indirect effects. The toll-like receptors 3 and 4 (TLR3 and TLR4) are activated by double-stranded RNA (dsRNA) as part of the innate immune response. In HIV-infected monocyte-derived macrophages (MDMs), TLR3/4 activation led to increased miR-155 expression, which in turn translationally repressed ADAM10, TNPO3, Nup153, and LEDGF/p75 (Swaminathan et al., 2013; Seddiki et al., 2014). As these HDFs are required for nuclear trafficking and integration of HIV, their repression via miR-155 negatively impacted viral replication. The targeting of TLRs was also recently connected to HIV replication via exosomes (Aqil et al., 2014). The viral accessory protein Nef influenced the secretion of at least 47 host miRNAs from exosomes, and target identification revealed an enrichment for pro-inflammatory and TLR transcripts (Aqil et al., 2014). As these miRNAs also function as cell non-autonomous signals for TLR activation, they may contribute to the ability of Nef-containing exosomes to trigger apoptosis in uninfected CD4^+^ T cells (Lenassi et al., 2010).

**Table 1:** Host miRNAs that have indirect and direct effects on HIV replication.

	miRNA name (miRBase v.18)	Cell type	Target	Effect on HIV replication	Reference
**Indirect**	miR-20a, miR-17-5p	Jurkat and CD8+ Depleted PBMCs	p300/CBP-associated factor	Suppression of Tat-mediated LTR activation	Triboulet et al. (2007)
	miR-1236	Monocytes	VprBP	Restricted Vpr-mediated modulation of cell cycle factors	Ma et al. (2014)
	miR-15a, miR-15b, miR-16, miR-93, miR-106b		PUR-α	Suppression of Tat-mediated LTR activation	Shen et al. (2012)
	miR-198		Cylin-T1	Suppressed HIV LTR activation	Sung and Rice (2009)
	miR-27b, miR-29b, miR-150, miR-223	Resting CD4+ T cells			Chang et al. (2012)
	miR-155	Monocyte-derived macrophages	ADAM10 TNP03 Nup153 LEDGF/p75	Restricted nuclear import of HIV Pre-integration complex	Swaminathan et al. (2012)
	miR-146a miR-888	HEK293 cells	AGO2 (endogenous small RNAs)	Inhibition of Gag multimerization	Chen et al. (2014)
	miR-132	Primary CD4+ T cells	MeCP2 and other	Enhanced replication/activation of latent virus	Chiang et al. (2013)
	miR-217	HeLa-derived MAGI cells	SIRT-1	Enhanced LTR activation replication	Zhang et al. (2012)
**Direct**	miR-133b, miR-138, miR-149, miR-326, miR-92a	42CD4 cells (HEK-derived)	HIV RNA	Post-transcriptional silencing of HIV RNA	Houzet et al. (2012)
	miR-28b, miR-125b, miR-150, miR-223 miR-382	CD4+T cells			Huang et al. (2007)
	miR-29a	Jurkat cells			Ahluwalia et al. (2008)
	miR-29b-3p, miR-33a-5p	MT2 and CD4+T cells			Reynoso et al. (2014)
	miR-423 miR-301a, miR-155	C8166 and TZM-bl cells			Whisnant et al. (2013)

Interestingly, many of the miRNAs enriched in exosomes (e.g., miR-125b, miR-29b, miR-223, and Let-7) have also been described as suppressors of HIV replication (Wang et al., 2009; Chiang et al., 2013; Whisnant et al., 2013). This suggests that HIV may actively and selectively utilize the exosomal secretion process to exclude miRNAs with antiviral activity. Alternatively, HIV may use exosomes to modulate the intracellular levels of RISC-free small RNAs that indirectly inhibit virion production (Chen et al., 2014). This latter observation is intriguing as it involves a novel mechanism in which host miRNAs decrease viral replication. HIV Gag proteins multimerize at the cell membrane to facilitate virus assembly, and this process is enhanced by the non-specific binding of Gag to host mRNAs (Muriaux et al., 2001; Jouvenet et al., 2009). Overexpression of miR-146a or miR-888 disrupted Gag multimerization leading to decreased virion production (Chen et al., 2014). The same effect was observed if AGO2 was depleted thereby leading to a general increase in endogenous small RNA levels. These findings also have implications for expression-based studies, as a comprehensive evaluation of variations in exosomal-miRNA secretion across different HIV strains and cell types has not been published. As a contributing factor to chronic T cell activation and the persistence of viral reservoirs (Jain et al., 2013), the control of miRNA export from exosomes by HIV definitely warrants further investigation.

The positive regulation of HIV replication by host miRNAs is far less well-described. Cellular miR-217 and miR-132, both targeting HDFs, lead to increased viral replication. In the first case, miR-217 suppressed SIRT-1 protein in HeLa-derived MAGI cells infected with HIV (Zhang et al., 2012). SIRT-1 deacetylates Tat thereby disrupting activation of the HIV LTR (Zhang et al., 2009), and overexpression of miR-217 releases the negative regulation. Similarly, in activated CD4^+^ T cells and Jurkat cells, up-regulation of miR-132 increased viral replication (Chiang et al., 2013). Notably, overexpression of miR-132 also modulated HIV latency by reactivating latent virus and delaying regression to latency following TNF-α treatment (Chiang et al., 2013). This was linked to a known miR-132 target, MeCP2, although the specific interactions remain unknown. Given the recent clinical success of miRNA-directed therapies targeting Hepatitis C Virus’ dependency on host miRNA-122 (Jopling et al., 2005; Shimakami et al., 2012), the exploitation of viral dependencies on host miRNAs may hold great promise for HIV as well. While our current repertoire of such dependencies remains limited, this exciting field of research along with the number of miRNAs able to regulate HIV replication should only increase in the coming years.

## Host miRNAs that Act on Virus Proteins to Affect HIV (Direct Effects on Viral Replication)

*In silico* approaches have been predominantly used to predict host miRNA binding sites within HIV transcripts, and many of these have subsequently been validated experimentally. Across multiple HIV clades, miR-29a and miR-29b were predicted to target HIV *nef* transcripts, while miR-149, miR-378, miR-324-5p were predicted to target *vpr*, *env,* and *vif* transcripts respectively (Hariharan et al., 2005). Exogenous expression of miR-29a did reduce Nef protein expression and subsequent viral replication in Jurkat cells, but this was not similarly true for miR-29b (Huang et al., 2007; Ahluwalia et al., 2008; Sun et al., 2012). In contrast, miR-29b suppressed HIV replication in 42CD4 cells (Houzet et al., 2012), MT2 cells and CD4^+^ T cells (Reynoso et al., 2014) although PAR-CLIP (a technique that directly links miRNAs to their cognate mRNA transcripts) revealed that miR-29b did not target *nef* transcripts (Whisnant et al., 2013; Reynoso et al., 2014). PAR-CLIP did reveal four putative miRNA-binding clusters for miR-423, miR-301a, miR-155, and miR-29a in the HIV genome, and the first three miRNAs were indeed able to directly bind to and negatively regulate HIV transcripts (Whisnant et al., 2013). However, miR-29a did not negatively regulate *nef* expression as previously observed (Huang et al., 2007; Ahluwalia et al., 2008; Sun et al., 2012). Taken together, these data once again hint at the difficulties in reliably identifying anti-HIV miRNAs. Compounded with the extremely high mutation rate inherent in HIV replication, and clear to any patient who has failed non-combination therapy, it is perhaps more wise to focus on those cellular miRNAs that *indirectly* inhibit HIV in our pursuit of novel anti-viral targets.

## HIV and RNAi Evasion Strategies

A discussion of cellular miRNA-mediated regulation of viral replication is not complete without touching upon counter strategies utilized by the pathogens themselves. The conservation of viral effectors known as viral suppressors of RNAi (VSR) or RNAi silencing suppressors (RSS) has been documented for many plant and animal viruses alike, suggesting that the host RNAi pathway may have been an important determinant of viral evolution in general (Li and Ding, 2006; Diaz-Pendon and Ding, 2008) and for HIV in particular (Qian et al., 2009; Coley et al., 2010; Hayes et al., 2011). HIV Tat has RSS activity as the viral protein abrogates Dicer functioning (Bennasser et al., 2005). By altering the Tat RNA binding domain via a lysine to alanine mutation (K51A), the RSS activity of Tat was abolished (Bennasser et al., 2005). Interestingly, a plant virus RSS, P19, could rescue HIV transcription following infection with a Tat K51A virus, although this study disputed that the RSS effects of Tat were via Dicer targeting (Qian et al., 2009). Viral Vpr and Nef may contribute to the RSS effects of Tat, as HIV strains deficient in these factors led to altered host miRNA expression profiles compared to wildtype virus (Hayes et al., 2011), and ectopic expression of Vpr or Nef down-regulated Dicer expression (Coley et al., 2010). Nef is also able to directly bind Ago2 leading to decreased cleavage of reporter transcripts (Aqil et al., 2013). Furthermore, wildtype Nef expression rescued ΔNef HIV infection, but Nef variants lacking Ago2 binding sites could not (Coley et al., 2010). HIV RNA has also been shown to have RSS activity. The host TAR RNA binding protein (TRBP) is an important factor in miRNA biogenesis as it binds Dicer in order to process pre-miRNAs into mature miRNA molecules (Haase et al., 2005). Depletion of TRBP thus negatively impacts endogenous RNAi-mediated silencing. HIV TAR RNA interacts with TRBP and this sequestration is thought to prevent interaction with Dicer thereby suppressing the RNAi pathway (Bennasser et al., 2005).

Another method employed by HIV to counter the cellular RNAi pathway involves secondary structures of the viral RNA itself. It has been postulated that the complex folding of HIV transcripts may represent a highly evolved strategy to evade regulation by host miRNAs (Watts et al., 2009). Indeed, miRNA-mediated suppression of *nef* transcripts was significantly impacted by increasing the length (and subsequent secondary structure) of these viral mRNAs (Sun et al., 2012). In addition, a shorter *nef* reporter transcript incapable of forming a predicted secondary loop, was highly suppressed by miR-29a and miR-29b. In addition, HIV transcripts exhibited a 100-fold higher refraction to RISC binding compared to host mRNAs (Whisnant et al., 2013). The finding that HIV RNA secondary structures renders them resistant to RNAi was first observed a decade ago (Westerhout et al., 2005). The initial effectiveness of a siRNAs targeting HIV RNA resulted in escape variants encoding nucleotide substitutions/deletions that led to altered RNA secondary structures and occlusion of the siRNA binding sites (Westerhout et al., 2005). Considering that significant variations in both transcript length and sequence composition have been documented between different HIV variants, even within a single infected individual (Péloponése et al., 1999; Bandaranayake et al., 2010), the efficacy of host miRNAs targeting these viral transcripts must vary as well. Furthermore these findings caution against the use of popular 3′ UTR reporter systems that do not accurately mimic the secondary structures of the RNAs for which they serve as proxies. As we already suggested, these findings do not bode well for miRNA-based therapies aimed at directly targeting viral RNA, but there is still vast hidden potential within the host miRNome for natural restriction of HIV replication.

## Perspectives

In this review we have discussed some of the mounting evidence that strongly suggests the host non-coding component is at the centre of a dynamic power struggle between virus and host, with each seeking to utilize the regulatory potential of non-coding RNAs to promote their own survival. We have covered the limited number host long non-coding RNAs that are manipulated by HIV, and it seems that the virus itself may also encode a lncRNA thereby enabling self-modulation of viral transcription (Saayman et al., 2014). This provides yet another interesting example whereby HIV manipulates cellular dark matter and closes the circular loop of ‘buildings’ that are shaped and then go on to shape those that reside in them.

Human immunodeficiency virus has also seemingly evolved a number strategies to selectively manipulate the host miRNA landscape, while at the same time protecting its own RNA transcripts within highly complex secondary structures that may occlude RISC-loaded miRNA binding (Sun et al., 2012; Whisnant et al., 2013). HIV viral proteins Tat, Vif, and Vpr have been shown to independently regulate the expression levels of a discrete subsets of host miRNAs (Hayes et al., 2011). While a few potential host miRNA regulators of HIV infection have been proposed by independent studies, a functional miRNome-wide interrogation of host miRNAs capable of modulating HIV replication remains conspicuously absent from the current literature.

A small number of miRNAs have been reported to be differentially expressed in both the PBMC population and plasma of elite controllers (Witwer et al., 2012; Reynoso et al., 2014). Additionally two of these miRNAs have also been shown to suppress HIV replication in primary CD4^+^ T cells thus suggesting a potential role for host non-coding RNAs in elite controller phenotypes. Greater insight into the general contribution of non-coding RNAs to the ‘elite control’ of HIV may therefore guide future therapeutic strategies and also expand our limited understanding of the endogenous functions of these molecules.

The non-coding ‘dark matter’ of the host represents a potentially abundant and relatively unexplored resource in terms of novel therapeutic approaches, while the practical adaptation of RNAi for therapeutic benefit remains in its infancy. Given the grand scale of the regulation mediated by the non-coding component of the human genome in so many clinically relevant disorders, including HIV infection, it would not be presumptuous to speculate that many future therapeutic breakthroughs may hinge on us shedding more light on the role of this ‘dark matter’ in infection.
